# Adaptive mutations in HA of avian H9N2 influenza viruses facilitate their transmission to swine

**DOI:** 10.1186/s13567-025-01678-7

**Published:** 2025-12-01

**Authors:** Jia Wang, Peiwen Chen, Qiwei Liu, Sixia Huang, Maocai Wu, Dan’er Wei, Wenshan Hong, Tommy Tsan-Yuk Lam, Huachen Zhu, Yi Guan

**Affiliations:** 1 Guangdong-Hong Kong Joint Laboratory of Emerging Infectious Diseases/Joint Laboratory for International Collaboration in Virology and Emerging Infectious Diseases, Joint Institute of Virology (STU/HKU), Shantou University, Daxue Road 243, Jinping District, Shantou, Guangdong People’s Republic of China; 2 State Key Laboratory of Emerging Infectious Diseases (SKLEID), School of Public Health, Li Ka Shing Faculty of Medicine, The University of Hong Kong, 5/F, 21 Sassoon Road, Pokfulam, Hong Kong SAR, China

**Keywords:** H9N2 avian influenza virus, cross-species transmission, swine infectivity, viral adaptation, hemagglutinin (*HA*) mutations

## Abstract

The cross-species transmission of avian H9N2 influenza viruses to swine increases the risk of viral adaptation to mammalian hosts. However, the mechanisms by which these H9 viruses can overcome the barriers posed by swine hosts have not yet been fully elucidated. In previous studies, we identified avian H9 strains exhibiting either infective or noninfective phenotypes in swine. Here, we investigated the role of surface genes in cross-species transmission by replacing the surface genes of noninfective strains with those of their infective counterparts. We demonstrated that the surface genes of the infective strains, particularly the hemagglutinin (*HA*) gene, restored infectivity in pigs for two previously noninfective strains. Surface genes from infective strains significantly increased viral replication efficiency in both CEF and PK15 cells, and recombinant viruses carrying these genes presented superior thermal stability. Amino acid sequence analysis of *HA* identified six critical residues (30T, 39A, 327R, 373K, 465K, and 490R) associated with infectivity in pigs. Avian H9 viruses bearing these swine-adaptive molecular signatures began emerging in terrestrial poultry before the 2000s and subsequently achieved dominance through widespread dissemination. These findings suggest that molecular changes in *HA* accumulated during the adaptation of avian H9 viruses in terrestrial poultry may drive the emergence of swine-infective strains. This study elucidates key molecular determinants that enable avian H9 viruses to infect swine and highlights the public health implications of prolonged H9N2 circulation in terrestrial poultry, which facilitates mammalian adaptation. These insights underscore the need for intensified surveillance of avian-to-swine influenza transmission dynamics.

**Supplementary Information:**

The online version contains supplementary material available at 10.1186/s13567-025-01678-7.

## Introduction

The first recorded instance of the H9 viruses in domestic poultry dates back to 1966 in turkeys in Wisconsin [1]. Since then, these viruses have been sporadically detected in both poultry and wild birds across North America. Owing to the migration of wild birds, H9 viruses were introduced into Korea and China during the 1990s. They subsequently became endemic in poultry throughout Asia, the Middle East, and North Africa over the following three decades [2–4]. Currently, H9 is one of the most widespread subtypes of low pathogenic avian influenza A virus globally, and it has also been identified in various mammalian species. To date, the H9 viruses have been detected in humans, swine, dogs, and minks [5–8]. In addition to cases exhibiting clinical signs of disease, retrospective serological surveys have revealed a relatively high rate of latent infection in human and swine populations [9–11]. The broad host range and mild virus‒host interactions associated with H9 enhance the survival of H9 viruses in nature and increase the likelihood of genetic exchange between H9 and other influenza virus subtypes [12, 13]. The internal genes of H9 viruses contribute to the emergence of H5N6, H7N9, H10N3, and H3N8 viruses that cause human infections [14–17].

The widespread prevalence of H9 viruses in domestic poultry increases the risk of interspecies transmission to swine, which are considered as the potential “mixing vessels” for influenza virus genes [18]. Since 1998, sporadic reports of swine infected with H9N2 viruses have been published [19, 20]. These repeated interspecies transmissions raise concerns that H9 viruses may adapt to humans through the accumulation of molecular changes in swine [21–23]. Additionally, there are concerns regarding the potential for the H9 virus to reassort with mammalian influenza viruses [24], which could generate novel pandemic strains, as coinfections of H9N2 with H1N1 and H3N2 have been identified in swine [13, 25, 26]. Experimental studies have demonstrated that the surface genes of H9 viruses exhibit high compatibility with those of human H1N1 and H3N2 viruses [27, 28]. Furthermore, reassortants containing surface genes from H9N2 viruses and internal genes from either H3N2 or H1N1 viruses have shown increased replication efficiency and transmissibility in pigs and ferrets [29]. These findings suggest that the surface genes of the H9N2 virus may possess the essential elements required for infecting swine; nevertheless, efficient aerosol transmission among mammals would require enhancement from internal genes [30, 31].

Surface genes play crucial roles in viral tropism, receptor binding, membrane fusion, and virion release. Amino-acid substitutions in these genes can lead to variants that overcome host restrictions. H9 field isolates exhibit spontaneous amino acid mutations, such as Q226L, T137I, I155T, H183N, and A190V in the *HA* (H3 mature *HA* numbering throughout the manuscript) [32–36]. These mutations have been experimentally linked to changes in binding preference with human receptors (SAα2,6-Gal) and an increase in viral replication in mammalian cells [33, 35, 37]. Amino acid substitutions that may facilitate the adaptation of H9 viruses in mammalian hosts were also investigated by serially passaging H9 viruses in animal models such as mice, guinea pigs, ferrets, and pigs. These studies revealed the potential involvement of *HA1*-D225G, *HA1*-Q227P, *HA2*-D46E, *NA*-27T, and *NA*-30T in mammalian adaptation [38–40]. However, for swine—an animal with a large population, some of which have access to poultry—the determinants of avian H9 viruses related to infectivity in pigs remain unclear. Phylogenetic analyses of the *H9* gene revealed three major clades within the Eurasian lineage: the Y439, G1, and Ck-Bei (or Y280) clades. In a previous study, we reported that viruses from the G1 and Y280 lineages were capable of infecting pigs, whereas those originating from the natural reservoir (Y439 lineage) remained noninfective [41]. This finding suggests that the evolution of the *H9* gene in land-based birds may have contributed to the introduction of H9 viruses to swine. In light of this, we investigated the role of surface genes in the interspecies transmission of the H9 virus to pigs by replacing the surface genes of infective and noninfective H9 viruses from aquatic and terrestrial sources, respectively.

## Materials and methods

### Viruses

Strains of A/Duck/Shantou/2030/2000 (ST2030, H9N1, Y439 clade), A/Duck/Jiangxi/7554/2007 (JX7554, H9N2, Y439 clade), and A/Chicken/Hong Kong/SSP177W/2009 (SSP177W, H9N2, Ck-Bei clade) were isolated from apparently healthy poultry. A/California/07/2009 (CA07, H1N1) was kindly provided by the World Health Organization Collaborating Centers for Reference and Research on Influenza (Atlanta, GA, USA). A/Gull/Maryland/704/1977 (ML704, H13N6) and A/Quail/Hong Kong/G1/1997 (G1, H9N2) were obtained from the repositories at the State Key Laboratory of Emerging Infectious Diseases and Center of Influenza Research, the University of Hong Kong. CA07 virus was passaged via Madin–Darby canine kidney (MDCK) cells, whereas the other viruses were propagated in 9- to 10-day-old embryonated chicken eggs. The viruses were stored at −80 °C until they were ready for use.

### Generation of recombinants and mutants via reverse genetics

**Figure 1:**
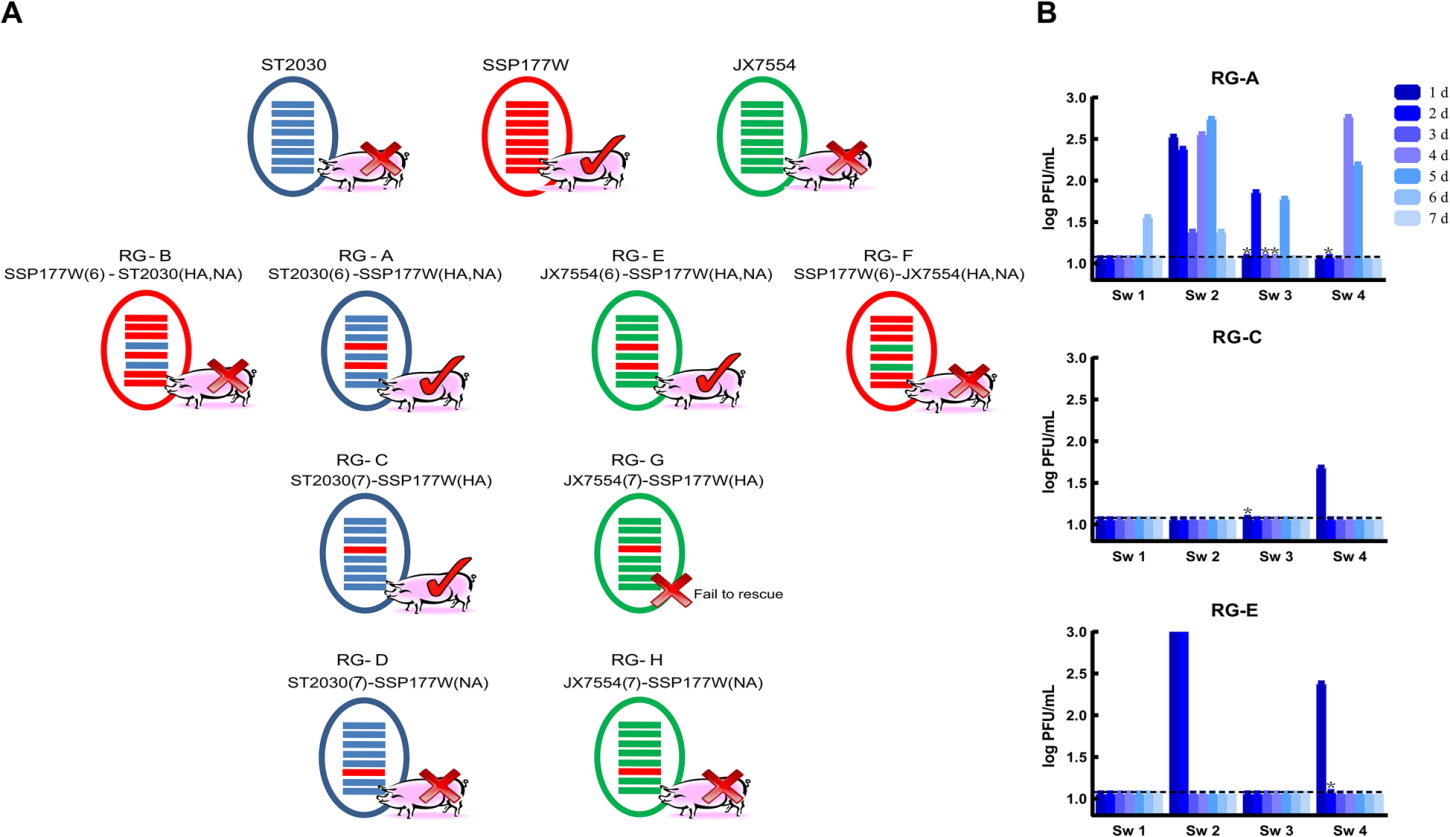
**Infectivity of the recombinants in pigs**.

Recombinants were constructed by replacing the surface genes either between ST2030 and SSP177W (recombinants designated reverse genetics [RG]-A, RG-B, RG-C, and RG-D) or between JX7554 and SSP177W (recombinants designated RG-E, RG-F, and RG-H). The gene constellation of each recombinant is shown in Figure 1. However, attempts to rescue RG-G, which was designed to replace the *HA* gene of JX7554 with that of SSP177W, were unsuccessful after three attempts. Mutants RG-SSP177W-M6 and RG-ST2030-M6, which carry specific amino acids at designated positions (as shown in Figure 5A), were constructed for the *HA* genes of SSP177W and ST2030. In brief, viral RNA was extracted via the QIAamp Viral RNA Mini Kit (Qiagen). cDNA was synthesized with the Uni-12 primer via the PrimeScript™ II 1st Strand cDNA Synthesis Kit (TAKARA). Eight full-length genes were amplified with Pfu Ultra^®^ II Fusion HS DNA Polymerase (Stratagene) and subsequently inserted into the pHW2000 plasmid (provided by Dr. R.G. Webster of St. Jude Children’s Research Hospital). The plasmids were sequenced, and only those with sequences identical to those of the parental virus were used to rescue the recombinants. Mutations in the *HA* genes of ST2030 and SSP177W were introduced via a site-directed mutagenesis kit from Trans Gene, Inc. Eight plasmids, each at a concentration of 1 μg, were incubated with 18 μL of Trans-LT1 (PANVERA, Madison, WI, USA) for 45 min. The plasmids were then transfected into cocultured monolayers of human embryonic kidney (HEK) 293T and MDCK cells. After 48–72 h, the culture supernatant was harvested and propagated from 9- to 10-day-old embryonated chicken eggs. The allantoic fluid containing the virus was collected to determine the median tissue culture infective dose (TCID_50_) and plaque-forming units (PFU). The identities of the recombinants and mutants were confirmed through sequencing.

### Cells, growth kinetics, and thermostability studies

MDCK, 293T, and pig kidney (PK15) cells were obtained from the American Type Culture Collection (ATCC). Primary chicken embryo fibroblast (CEF) cells were isolated from 8-day-old specific pathogen-free (SPF) chicken embryos. MDCK cells were cultured in Eagle’s minimal essential medium (MEM) supplemented with 10% fetal bovine serum (FBS), while 293T, PK15, and CEF cells were cultured in Dulbecco’s modified Eagle’s medium (DMEM) supplemented with 10% FBS. For the growth kinetics study, we applied ST2030, JX7554, SSP177W, and the corresponding recombinants to the CEF and PK15 cells at a multiplicity of infection (MOI) of 0.001. In the CEF and PK15 culture systems, trypsin was incorporated at concentrations of 0.1% and 0.15%, respectively. At 12, 24, 48, and 72 h post-inoculation, the supernatants were collected for TCID_50_ titration. The thermostability of ST2030, JX7554, and the corresponding recombinants was assessed by incubating the virus continuously for 24 h at 35 °C, 37 °C, 39 °C, 41 °C, and 43 °C. The virus was then applied to MDCK cells for TCID_50_ titration at 0, 8, 16, and 24 h post-incubation. Three independent experiments were performed.

### Animals and experimental infections

In total, 36 domestic piglets aged 6–8 weeks were used in the experiment. Prior to infection, the pigs were confirmed to be seronegative (with hemagglutination inhibition [HI] titers < 1:40) for the H1, H3, and H9 viruses through HI assays. Nasal swabs were collected and subsequently inoculated into MDCK cells and embryonated chicken eggs to confirm the influenza-free status. For each recombinant and mutant strain, four pigs were intranasally inoculated with 10^7^ PFU of the virus diluted in 1 mL of phosphate-buffered saline (PBS). Nasal swabs were collected daily from 1 to 8 days post-inoculation (dpi), and nasal virus secretion was determined via plaque assays. Rectal temperature and signs of disease were recorded from 0 to 8 dpi. Serum samples were collected at 14 and 28 dpi for detection of antibodies against H9 viruses via HI and viral microneutralization (MN) assays according to protocols recommended by the World Health Organization (WHO).

### Receptor binding preference studies

Chicken and horse erythrocytes were used for hemagglutination (HA) assays to investigate the receptor-binding preferences of ST2030, JX7554, SSP177W, CA07, G1, ML704, and the recombinants. Chicken red blood cells (CRBCs) were suspended in phosphate-buffered saline (PBS) to a final concentration of 0.55%. Horse erythrocytes (HRBCs) were suspended in PBS supplemented with 0.5% bovine serum albumin to a final concentration of 0.7%. For the hemagglutination assay, viruses were serially twofold diluted in a 96-well plate. Subsequently, 50 µL of each diluted virus was then mixed with an equal volume of the respective erythrocyte suspension, and the mixture was incubated at 4 °C to allow complete hemagglutination. The CRBC assay was conducted in U-bottom microtiter plates, whereas the HRBC assay was performed in V-bottom plates. All tests were performed in duplicate.

### Virus elution assay

The HA assay was performed by using viruses with an HA titer of 1:64. Starting from the HA titer of 1:64, the viruses were subjected to serial twofold dilutions. Subsequently, 50 μL of the diluted virus solution was mixed with an equal volume of 0.55% CRBCs in a U-bottom microtiter plate. The plate was held at 4 °C for 30 min for virus adsorption to the CRBCs and then incubated at 37 °C for 12 h continuously. The HA titer was monitored at 30 min, 1 h, and then every 2 h after incubation. A decrease in the HA titer, which coincided with neuraminidase (NA)-mediated virus elution, was observed. The assay was performed in three independent experiments.

### Sequence and protein structure analysis

*HA* sequences of avian H9 (*n* = 8003), swine H9 (*n* = 42), and human H9 (*n* = 84) viruses were obtained from the GISAID EpiFlu™ Database and analyzed with those of SSP177W, ST2030, and JX7554 (BioEdit software, version 7.7.1.0; Tom Hall, Ibis Biosciences, Carlsbad, CA, USA). The contribution of the GISAID sequence is acknowledged in Additional file 1. The amino acid sequences of *HA1* and *HA2* of ST2030 were uploaded to the SWISS-MODEL website (Swiss Institute of Bioinformatics, University of Basel) for the protein structure modeling [42]. DeepView (version 4.1, Swiss Institute of Bioinformatics) was used to visualize the structure and analyze the accessibility of the target amino acids.

### Statistical analysis

The data were analyzed via analysis of variance (ANOVA) and Tukey’s comparisons test via GraphPad Prism software (version 9). When compared with ST2030 and JX7554, respectively, the recombinant strains with SSP177W surface genes were considered statistically significant differences if *p* < 0.05, and highly statistically significant differences if *p* < 0.01.

## Results

### Recombinants carrying surface genes from SSP177W were endowed with infectivity in pigs.

Our prior investigation demonstrated that intranasal administration of SSP177W in swine results in viral shedding within the nasal cavity. The shedding titers were observed to range between 10^2^ and 10^4^ PFU/mL during days 2–6 post-inoculation. Additionally, H9-specific neutralizing antibodies were identified in three pigs, with titers of 1:64, 1:64, and 1:128, respectively. In contrast, no virus was detected in pigs inoculated with either ST2030 or JX7554 [41]. In this study, we exchanged the surface genes of SSP177W and ST2030 and those of SSP177W and JX7554 to investigate the role of surface genes in the infectivity of the H9 virus in pigs. We found that pigs inoculated with RG-A and RG-E, i.e., the reassortant viruses with both *HA* and *NA* genes from SSP177W, exhibited virus excretions in the nasal cavity. All pigs in the RG-A group had nasal shedding, with two pigs (Sw2 and Sw3) shedding continuously for 5 days (Figure 1B). The rectal temperatures of these four pigs increased from a baseline of 39.40 ± 0.72 °C (−3 to 0 dpi) to 41.38 ± 0.34 °C (1 to 5 dpi) and returned to normal levels after 6 dpi. All pigs in the study presented a runny nose, with Sw3 displaying the most severe symptoms. Each pig in the RG-A group seroconverted (HI ≥ 1:40), and two pigs (Sw2 and Sw4) were found to have neutralizing antibodies (MN ≥ 1:40) (Table 1). In the RG-E group, Sw2 and Sw4 shed the virus for 2 days after inoculation. Seroconversion was observed in these two pigs, and Sw4 generated H9 neutralizing antibodies. Additionally, Sw1, Sw2, and Sw3 of the RG-E group presented runny nose symptoms, but their rectal temperature remained normal after virus inoculation. In contrast to the RG-A and RG-E groups, pigs inoculated with RG-B and RG-F, which contained the internal genes of SSP177W and the surface genes of ST2030 and JX7554, respectively, yielded negative results in both nasal swab titration and serum antibody detection. Although runny noses were occasionally observed in pigs from these two groups, their temperature remained normal throughout the experiment.

**Table 1:** **Seroconversion of pigs inoculated with the recombinants**

Group		Hemagglutination inhibition (HI) titer							Microneutralization (MN) titer						
		14/21 dpi							14/21 dpi						
		RG-A	RG-B	RG-C	RG-D	RG-E	RG-F	RG-H	RG-A	RG-B	RG-C	RG-D	RG-E	RG-F	RG-H
RG-A	Sw1	**40/80**							**−/10**						
	Sw2	**320/320**							**80/160**						
	Sw3	**80/40**							**−/10**						
	Sw4	**320/80**							**80/80**						
RG-B	Sw1		**10/−**^a^							**20/−**					
	Sw2		**−/−**							**10/10**					
	Sw3		**−/−**							**−/−**					
	Sw4		**−/−**							**10/−**					
RG-C	Sw1			**40/20**							**−/−**				
	Sw2			**10/−**							**−/−**				
	Sw3			**−/−**							**−/−**				
	Sw4			**80/80**							**10/10**				
RG-D	Sw1				**−/−**							**−/−**			
	Sw2				**−/−**							**−/−**			
	Sw3				**−/−**							**−/−**			
	Sw4				**−/−**							**−/−**			
RG-E	Sw1					**20/20**							**−/−**		
	Sw2					**160/160**							**20/20**		
	Sw3					**10/−**							**−/−**		
	Sw4					**320/320**							**40/40**		
RG-F	Sw1						**−/−**							**−/−**	
	Sw2						**−/−**							**−/−**	
	Sw3						**−/−**							**10/−**	
	Sw4						**−/−**							**−/−**	
RG-H	Sw1							**−/−**							**−/−**
	Sw2							**−/−**							**−/−**
	Sw3							**−/−**							**−/−**
	Sw4							**−/−**							**−/−**

^a^HI or MN titer < 10

*An antibody titer of 40 or greater is regarded as indicative of seroconversion. dpi*:days post-inoculation

In the recombinant groups containing either the *HA* or the *NA* of SSP177W, we found that the inoculation of RG-C, which possessed the *HA* gene from SSP177W and the remaining genes from ST2030, resulted in a low level of nasal shedding in Sw3 and Sw4 at 1 dpi (Figure 1B). Two pigs (Sw1 and Sw4) in this group seroconverted, but no neutralizing antibodies were detected (Table 1). In the RG-D and RG-H groups, neither viruses nor antibodies were detected, and all pigs remained in normal physical condition throughout the experiment.

### Recombinants harboring surface genes from SSP177W demonstrated superior replication in CEF and PK15

**Figure 2:**
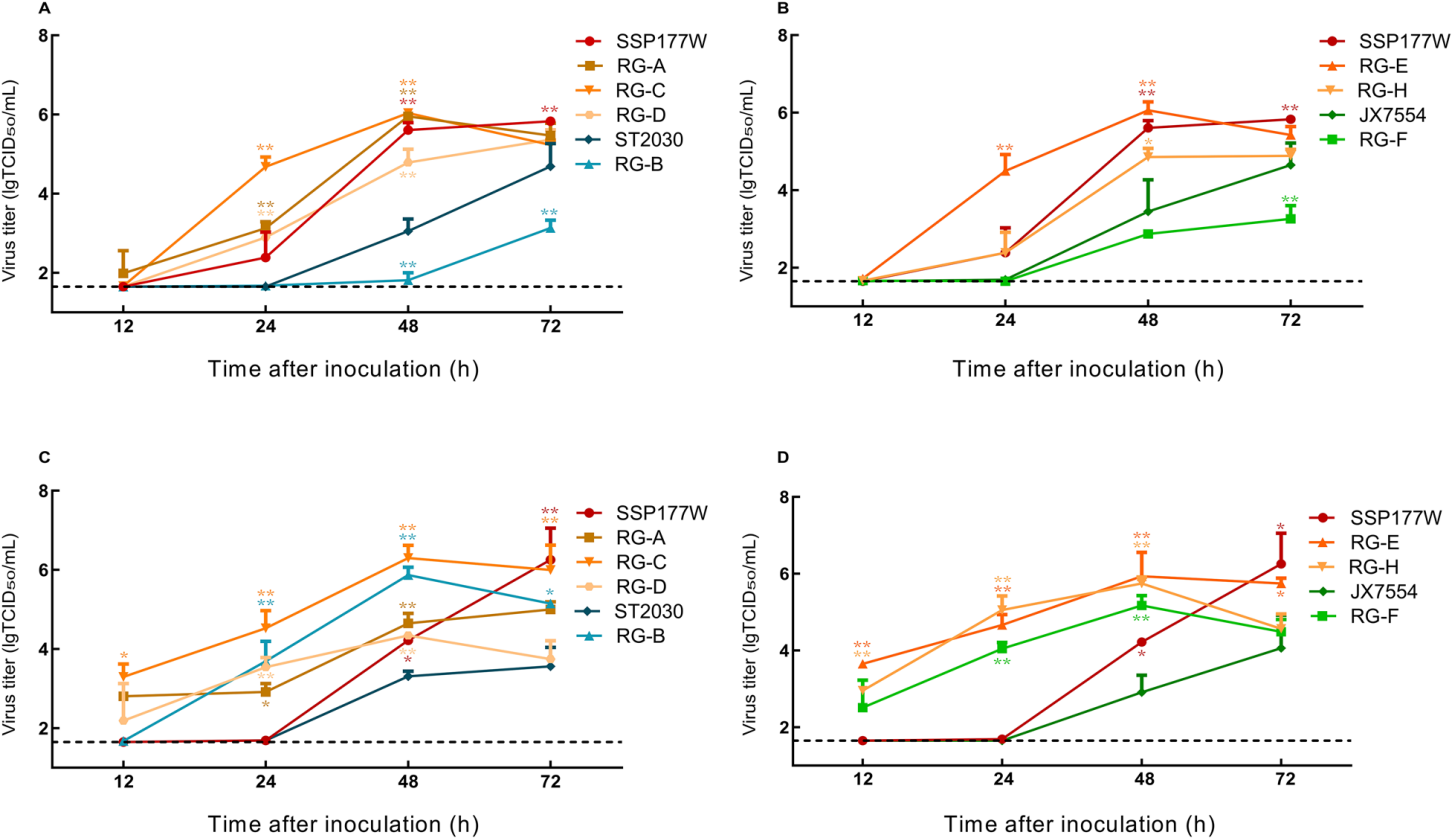
**Growth kinetics of the recombinants in the CEF and PK15 cells**.

In CEF cells, SSP177W demonstrated significantly better replication efficiency than ST2030 and JX7554 did (*p* < 0.01) (Figure 2A, B). RG-A, RG-C, and RG-D, which carry either one or two surface genes from SSP177W, achieved higher replication efficiency than the prototype ST2030 (Figure 2A). At 24 hpi, RG-C with only *HA* from SSP177W presented a significant growth advantage over SSP177W and at 48 hpi over ST2030 (*p* < 0.0001). In contrast, RG-B, which has internal genes from SSP177W and surface genes from ST2030, exhibited poor replication. The exchange of *HA* and/or *NA* between SSP177W and JX7554 resulted in a similar pattern of change in replication efficiency (Figure 2B). The paired *HA*–*NA* from SSP177W provided a significant growth advantage to RG-E, whereas the surface genes from JX7554 decreased the growth of RG-F in CEF. These results suggest that surface genes determine the replication efficiency of the recombinants in chicken cells.

In PK15 cells, SSP177W also demonstrated a growth advantage over ST2030 and JX7554 (*p* < 0.05). The surface genes from SSP177W were found to confer better growth to recombinant viruses RG-A and RG-E relative to their counterparts with surface genes from ST2030 and JX7554, respectively (Figures 2C, D). Additionally, improved growth was observed in RG-C, RG-D, and RG-H, which contained a single surface gene from SSP177W. Interestingly, RG-B and RG-F, which carry internal genes from SSP177W and surface genes from ST2030 or JX7554, presented better replication efficiency than SSP177W. These findings suggest that the internal genes of SSP177W function well with the surface genes from ST2030 and JX7554 in PK15.

### The surface genes of SSP177W conferred better thermostability to the recombinants

**Figure 3:**
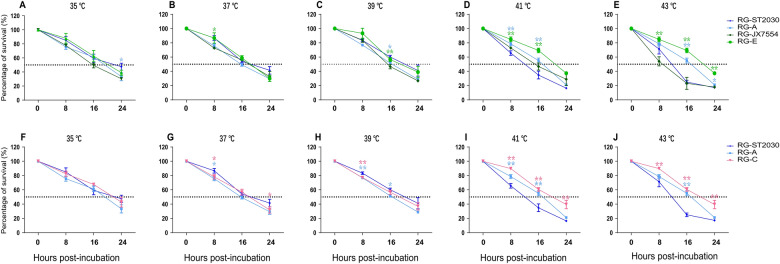
**Thermostability of the recombinants**.

The temperature range in the porcine respiratory tract is typically between 36 °C (nasal cavity) and 42 °C (pulmonary artery) [43]. To test the thermostability of the viruses, we incubated the virus aliquots at temperatures of 35, 37, 39, 41, and 43 °C and then titrated the aliquots that were collected at 0, 8, 16, and 24 h post-incubation. In general, all strains presented a similar decrease in titer when incubated at temperatures below 40 °C (Figures 3 A–C, F–H). The prototype viruses (ST2030 and JX7554) were able to maintain 50–60% infectivity after incubation at 40 °C for 16 h, while incubation at 41 °C and 43 °C had an obvious negative effect on viral infectivity. However, the infectivity of the recombinants (RG-A and RG-E), which possessed the *HA* and *NA* genes from SSP177W, was better than that of their prototype viruses at temperatures above 40 °C (Figure 3D, E). At 16 h post-incubation, RG-A and RG-E were 20% more infective than their prototype viruses, and RG-E showed a marked survival advantage at high temperatures.

To further investigate whether the *HA* gene contributes to survival at relatively high temperatures, we analyzed the thermostability of RG-C, which was constructed with the *HA* of SSP177W and the remaining genes of ST2030. RG-C had better thermostability than RG-A and ST2030 (Figures 3I, J), suggesting that the *HA* gene from SSP177W alone is sufficient to improve the thermostability of the reassortant.

### Receptor binding preference of SSP177W

By testing the hemagglutination ability of the virus with chicken and horse erythrocytes, we compared the receptor binding preferences of SSP177W, ST2030, JX7554, and the recombinants with those of a panel of viruses with different host adaptation implications. H9 viruses from lineages that are prevalent in terrestrial poultry, including SSP177W, barely bind to horse red blood cells, which possess only α2,3-sialic-acid-linked receptors (Table 2). However, hemagglutination was well observed with these viruses when CRBCs possessing two types of sialic-acid-linked receptors were used. The hemagglutination response pattern of these H9 viruses was similar to that of CA07, which is capable of infecting humans. In contrast, ST2030 and JX7554 hemagglutinated horse erythrocytes as did ML704, showing a similar binding preference to avian influenza viruses circulating in the natural reservoir. Interestingly, recombinant viruses carrying the SSP177W, ST2030, and JX7554 *HA* genes exhibit red blood cell binding preferences that align with those observed in the corresponding *HA* parental viruses.

**Table 2:** **Receptor binding preferences of the viruses**

Host type	Strain	Subtype	HA with	
			CRBC	HRBC
Mammal	A/California/07/2009	H1N1	32	0
Terrestrial poultry	A/Chicken/Hong Kong/SSP177W/2009	H9N2	2048	0
Terrestrial poultry	A/Quail/Hong Kong/G1/1997	H9N2	1024	0
Aquatic poultry	A/Duck/Shantou/2030/2000	H9N1	32	8
Aquatic poultry	A/Duck/Jiangxi/7554/2007	H9N2	64	64
Aquatic birds	A/Gull/Maryland/704/1977	H13N6	256	64
	RG-A: ST2030(6)-SSP177W(*HA*,*NA*)	H9N2	256	0
	RG-B: SSP177W(6)-ST2030(*HA*,*NA*)	H9N1	128	8
	RG-C: ST2030(7)-SSP177W(*HA*)	H9N1	512	0
	RG-D: ST2030(7)-SSP177W(*NA*)	H9N2	64	1
	RG-E: JX7554(6)-SSP177W(*HA*,*NA*)	H9N2	256	0
	RG-F: SSP177W(6)-JX7554(*HA*,*NA*)	H9N2	256	64
	RG-H: JX7554(7)-SSP177W(*NA*)	H9N2	32	0

The name of each reassortant virus is followed (after the colon) by its gene composition. In the format “XX(Y)-XX(genes)", Y refers to the number of internal genes from the first parent (e.g., ST2030), while the second parent (e.g., SSP177W) specifies the inserted *HA*/*NA* surface genes.

### The NA of SSP177W reduced the efficiency of erythrocyte elution in recombinants containing the ST2030 backbone

**Figure 4:**
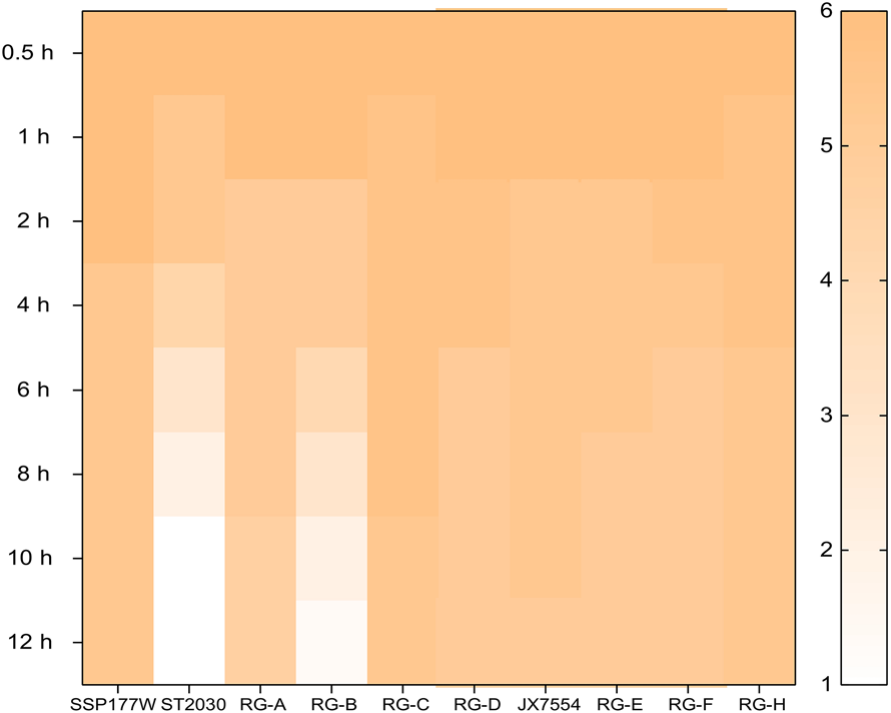
**Elution efficiency of the recombinants**.

In the influenza virus replication cycle, HA is responsible for binding to the receptor on the host cell and initiating infection, while the role of NA is to release the progeny virus by cleaving the bonds between HA and sialic acid on the cell membrane. In the porcine infection experiment, the infectivity of RG-C was markedly lower compared with that of RG-A, whereas RG-D exhibited a complete inability to infect pigs. These findings indicate that the compatibility between the functions of NA and HA plays a role in determining viral infectivity in porcine hosts. Here, we investigated the compatibility between the *HA* and *NA* genes by assessing the elution efficiency of the recombinants from the CRBCs. Among all the strains tested, ST2030 was found to have a remarkably high elution efficiency (Figure 4). The HA titer of ST2030 started to decrease at 1 hpi and declined rapidly over the next 12 h. In contrast, SSP177W and JX7554 bound well to the CRBC, and their HA titers remained stable throughout the test. Recombinant viruses RG-A, RG-B, and RG-F, which respectively harbor the paired *HA* and *NA* gene segments derived from SSP177W, ST2030, and JX7554, demonstrated red blood cell dissociation patterns that were congruent with those observed in their corresponding parental viruses. When comparing elution efficiency between SSP177W and RG-C, and between ST2030 and RG-D, the NA from ST2030 appeared more efficient than that from SSP177W at releasing virus from CRBCs. The elution efficiencies of JX7554, RG-H, and SSP177W were similar, suggesting that the *NA* genes of JX7554 and SSP177W have similar enzymatic capacities.

### Screening for molecular markers that may be associated with infectivity in pigs

To search for potential molecular signatures of H9 viruses that exhibit infectivity in pigs, we compared the *HA* amino acid sequences of ST2030 and JX7554 with those of four avian H9 viruses known to infect pigs [41]. Overall, 16 positions with different amino acids between the viruses capable and incapable of infecting pigs were found (Additional file 2). Further analysis was performed using sequences of swine H9 (*n* = 42). We found that 9 out of the 16 amino acid sites categorized as group B either possessed amino acids situated in positions that were not easily accessible or presented similar side chain motifs (Table 3). Consequently, substitutions at these sites are likely to have a minor effect on the functionality of HA. In contrast, group A presented distinct amino acid characteristics between H9 viruses with the capacity to infect pigs and those lacking this capability. Notably, the presence of arginine at position 327 in viruses exhibiting infectivity in pigs resulted in the introduction of an extra basic amino acid at the P4 position, which lies immediately upstream of the cleavage site. The presence of multiple basic amino acids within the connecting peptides may alter the virus’s sensitivity to trypsin, thereby enhancing its infection and replication efficiency. Furthermore, the positioning of the remaining five amino acids within the HA protein suggests that alterations in their properties could influence the interaction between HA1 and HA2, thereby affecting the process of membrane fusion (Additional file 3).

**Table 3:** **Potential molecular signatures of the H9 viruses that infect pigs**

Group	Position^a^	Avian H9 viruses^b^		Swine H9 viruses	Classification of amino acids	Location in the HA protein^c^
		Noninfective to pigs	Infective to pigs			
A	30	I	T	40T/1V/1S	I-NP, Aliph T-P	HA1-fusion domain
	39	T	A	41A/1T	T-P A-NP, Aliph	HA1-fusion domain
	327	A	R	40R/1A/1V	A-NP, Aliph R-Pos	Connection peptide
	373 (43)	E	K	38K/4R	E-Neg K-Pos	HA2-A helix
	465 (135)	N	K	41K/1N	N-P K-Pos	HA2-E to F domain
	490 (160)	Q	R	40R/1Q/1I	Q-P R-Pos	HA2-H helix
B	71	V	I	41I/1V	V and I-NP, Aliph	HA1-esterase subdomain low accessibility
	114	F	L	41L/1F	F-NP, Arom L-NP, Aliph	HA1-110 helix inner of the trimer
	197	K	R	40R/1K/1N	K and R-Pos	HA1-190 helix
	84	K	R	35R/2K/4G/1I	K and R-Pos	HA1-esterase subdomain low accessibility
	96	I	V	33V/2I/7T	I and V-NP, Aliph	HA1-esterase subdomain
	119	N	S	37S/4R/1K	N and S-P	HA1-130 loop low accessibility
	156	H	Q	39Q/3H	H-Pos Q-P	HA1-130 loop low accessibility
	369 (39)	E	D	22D/18E	E and D-Neg	HA2-A helix
	386 (56)	I	V	40V/2I	I and V-NP, Aliph	HA2-A helix
	405 (75)	A	T	34T/8A	A-NP, Aliph T-P	HA2-B loop inner of the trimer

^a^H3 mature *HA* numbering; the figure enclosed in parentheses indicates the corresponding *HA2* number

^b^Avian H9 viruses noninfective to pigs: A/Duck/Shantou/2030/2000, A/Duck/Jiangxi/7554/2007; avian H9 virus infective to pigs: A/Chicken/Hong Kong/SSP177W/2009, A/Chicken/Hong Kong/YU341/2008, A/Chicken/Hong Kong/NT449/2007, and A/Chicken/Hong Kong/NT10/2011

^c^HA protein structure modeling was conducted with the amino acid sequence of ST2030 on SWISS-MODEL (University of Basel) and analyzed with Swiss-PdbViewer 4.0.4

NP: nonpolar, P: polar charged, Neg: negatively charged, Pos: positively charged, Aliph: aliphatic R group, Arom: aromatic R group

### Amino acid substitutions at six potential determinant sites reversed the infectivity of ST2030 and SSP177W

**Figure 5:**
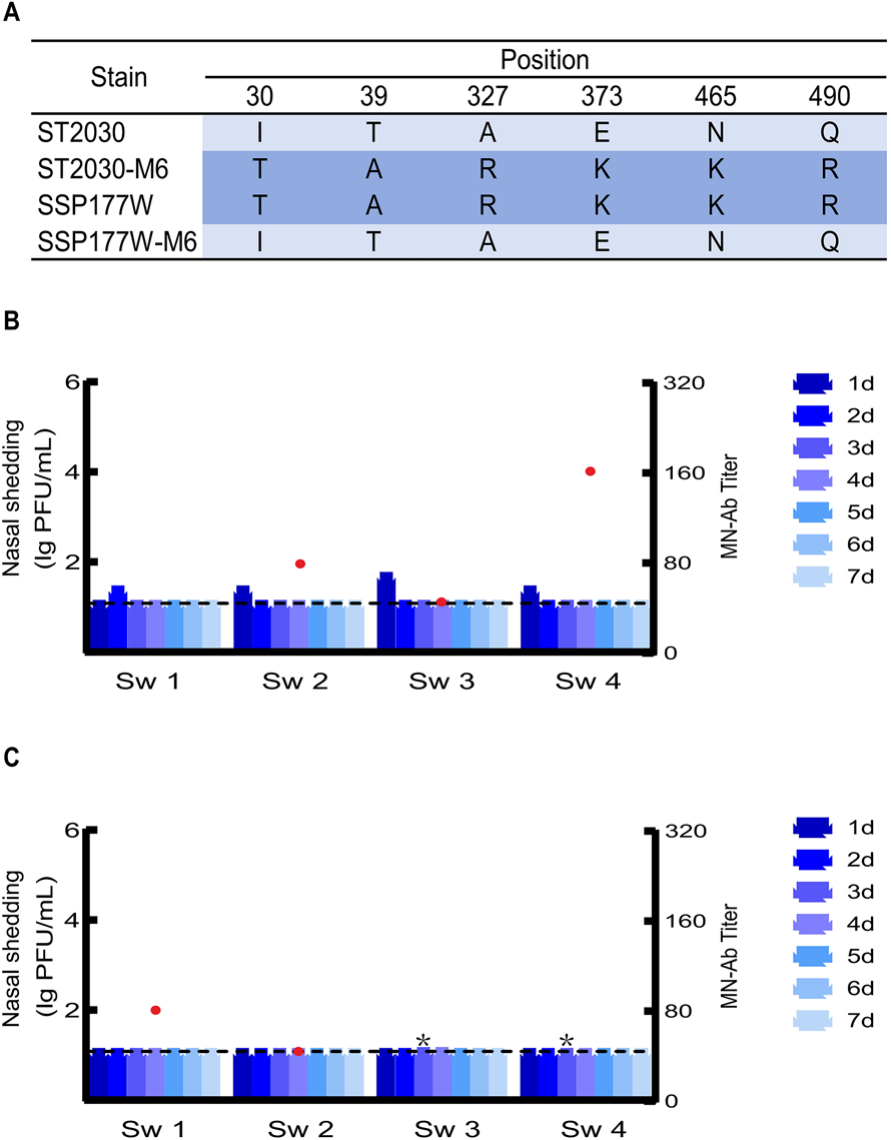
**Infectivity of the ST2030 and SSP177W mutants in pigs**.

As presented in our previous study, pigs inoculated with ST2030 exhibited neither viral shedding nor the presence of microneutralization antibodies (MN-Ab) at titers exceeding 1:40. In contrast, inoculation with SSP177W led to nasal viral shedding in four pigs and elicited the production of H9-specific neutralizing antibodies [41]. To test the effects of the potential determinants, RG-ST2030-M6 and RG-SSP177W-M6 were engineered to carry amino acids differing from each other at positions 30, 39, 327, 373, 465, and 490 in the *HA* and were intranasally inoculated into pigs (Figure 5A). Remarkably, positive shedding was detected in all pigs inoculated with RG-ST2030-M6. Although the shedding titer was low and persisted for only 1–2 dpi, seroconversion, as reflected by the MN titer, was detected in three pigs at 21 dpi, with titers of 1:40, 1:80, and 1:160 (Figure 5B). In contrast, nasal shedding was barely detectable in pigs inoculated with RG-SSP177W-M6 (Figure 5C), indicating that the amino acid substitutions significantly impaired the infectivity of RG-SSP177W-M6 in pigs.

### Distribution dynamics of six potential determinants of H9 viruses detected in poultry, swine, and humans

**Figure 6:**
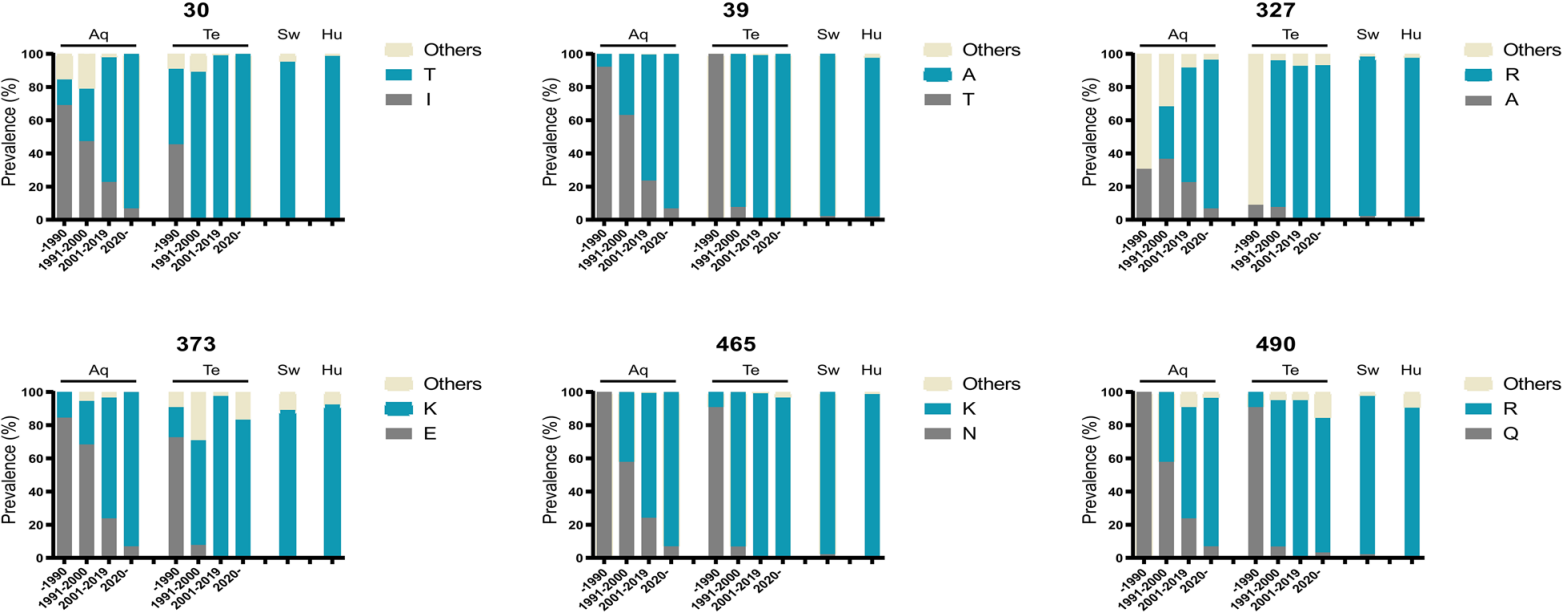
**Dynamics of the prevalence of amino acids at designated positions**.

To investigate whether the amino acids at these positions were selected during the evolution of H9 viruses in nature, we retrieved H9 sequences from the GISAID EpiFlu™ database and grouped them by time (before 1990, from 1991 to 2000, from 2001 to 2019, and after 2020) and by host (aquatic birds, terrestrial birds, swine, and humans). The percentages of amino acids at the above positions in viruses circulating in a given time period and host are summarized in Figure 6. Except at position 327, viruses with the same amino acids as those in ST2030 and JX7554 (gray columns) were predominant in both aquatic and terrestrial birds before 1990. During the 1990s, the proportion of viruses that possessed these amino acids decreased, and the proportions of viruses with 30T, 39A, 373K, 465K, and 490R (blue columns) increased. The downward trend was more pronounced for terrestrial H9 viruses. During the period of 2001–2019, more than 90% of terrestrial H9 viruses and 50% of aquatic H9 viruses contained 30T, 39A, 373K, 465K, and 490R. In contrast, 30I, 39T, 373E, 465N, and 490Q were conserved in the minority of aquatic H9 viruses, and they were rarely found in terrestrial strains after 2000. At position 327, alanine was one of many variants in the early stages of H9 establishment in poultry. However, all the variants were gradually replaced by arginine, which became predominant in terrestrial H9 viruses after 2001. These findings revealed the selection of amino acids during the evolution of H9 viruses in nature, especially during the adaptation of the virus in terrestrial birds. Importantly, these signatures were also observed in the majority of the human and porcine H9 isolates, suggesting that these changes may be important for adaptation in mammalian hosts.

## Discussion

Many subtypes of avian influenza viruses can occasionally spill over to swine but not as frequently as H9 [44, 45]. A serological survey of 534 swine farms in China revealed that 10.3% of pigs were positive for H9 antibodies [9]. In humans, the number of infected cases has dramatically increased over the last 4 years, bringing the total number of infected cases since 2015 to 133 [5]. The frequent recurrence of the interspecies transmission of the H9 virus to mammals suggests that there may be some factors on the *HA* gene that contribute to the disruption of mammalian host restriction. In porcine airways, sialic acid α2,6-galactose (SAα2,6-Gal) receptors are abundantly expressed on the epithelium from the nasal cavity to the lobar bronchus, whereas the expression of SAα2,3-Gal receptors increases gradually along the terminal bronchiole toward the alveoli [46, 47]. This biological structural basis creates a bottleneck for interspecies transmission: Viruses with a preference for SAα2,6-Gal receptor binding have a greater chance of adhering to and invading the upper respiratory tract. This explained the detection of nasal shedding in pigs inoculated with recombinants carrying the *HA* gene from SSP177W, which had a receptor binding preference similar to that of the human pandemic strain CA07. Molecular surveillance data have shown that an increasing proportion of H9N2 viruses with binding affinity for SA α2,6-linked receptors are circulating in terrestrial birds [48–51], suggesting that more H9 viruses have achieved the potential to recognize and bind to mammalian receptors.

As shown in many studies, HA-226L and HA-228G are not the exclusive determinants related to the ability of H9 viruses to bind to SA α2,6-linked receptors [22, 33]. In the receptor-binding pocket, A190V and D225G, generated during the serial passage of H9 virus in porcine airway epithelial cells and in pigs, were found to have a broader receptor-binding spectrum and better replication efficiency [21–23]. However, in this study, the infective strain (SSP177W) and noninfective strains (ST2030 and JX7554) all possessed 190A and 225G, suggesting that these two positions may not be critical factors for infecting pigs. HA-197, which is located at the terminus of the 190 helix, was found to have different amino acids between groups capable and incapable of infecting pigs. Given the structural similarity between arginine and lysine, the K197R mutation was not generated in the present study, leaving its potential role in porcine infection to be further explored.

In addition to the receptor-binding domain, all potential infection molecular markers identified in this study clustered in the HA stem region. A327R introduced an additional basic amino acid at position P4 before the cleavage site. During the evolutionary history of H9 in nature, nil-, mono-, di-, and even tri-basic amino acid connection motifs have been observed [52]. Previous research has demonstrated that H9 viruses possessing di-basic amino acids continue to exhibit low pathogenicity in chickens [53]. However, in the present study, the HA-327R mutation, in conjunction with five additional mutations, facilitated the ability of RG-ST2030-M6 to infect pigs. Conversely, the reversion of these mutations resulted in the loss of infectivity of RG-SSP177W-M6 in pigs. Similarly, an alanine-to-serine substitution at the P5 cleavage site was found to improve the virulence of the H9 virus in mice [54], suggesting that a change in cleavage efficiency may influence the introduction of the H9 virus into mammals. As the emergence of the furin recognition motif (R-X-R/K-R) is critical for the generation of highly pathogenic avian influenza viruses, the molecular evolution at the cleavage site of the H9 virus should be closely monitored. In addition to A327R, the other five potential determinants could be involved in the interaction between HA1 and HA2. Although no molecular marker in this region has been identified in experiments using porcine in vivo or in vitro models, serial adaptation of an H9N2 virus in guinea pigs revealed that a D46E substitution in HA2 could contribute to adaptation. D46E improved the thermostability of the virus and promoted aerosol transmission when it was combined with HA1-Q227P and NP-E434K [38]. This result is consistent with what we found in this study: Six molecular changes in the stem region of HA altered the infectivity of SSP177W and ST2030, and reassortants with *HA* from SSP177W presented the best thermostability. This is the first study in which amino acid substitutions in the HA stem region were experimentally linked to the infectivity of H9 viruses in pigs.

The prevalence dynamics of these amino acids over the past three decades have shown that these substitutions have been selected by long-term adaptation in terrestrial birds. To date, the vast majority of terrestrial H9 viruses harbor the amino acid residues 30T, 39A, 327R, 373K, 465K, and 490R; however, the functions associated with these residues remain to be elucidated. The long-term prevalence and adaptation of H9 viruses in terrestrial birds has generated and accumulated substitutions that contribute to their infectivity in swine. Moreover, amino acids at these six positions are also present in more than 90% of H9 human viruses, although their role in human infection remains to be investigated. Our findings highlight the risk of the continued prevalence and evolution of H9 viruses in terrestrial birds and urge molecular surveillance of H9N2 viruses for early pandemic preparedness.

This study investigates the influence of surface genes of H9 subtype avian influenza viruses on their infectivity in swine; however, the contribution of the viruses’ internal genes in this process remains unexamined. In replication efficiency assays conducted with PK15 cells, the SSP177W strain demonstrated a distinctive pattern characterized by delayed replication during the early phase (12–24 h) followed by a pronounced replication surge in the late phase (48–72 h). Conversely, the recombinant virus RG-B, which harbors the internal genes of SSP177W combined with the surface genes of ST2030, as well as RG-F, containing the surface genes of JX7445, both exhibited significantly enhanced replication efficiency during the early phase relative to the parental SSP177W strain. This phenomenon may be partially attributed to the necessity for wild-type viruses to undergo a period of adaptation to the cellular microenvironment following their isolation and propagation in chicken embryos. Furthermore, it implies that, beyond the role of surface genes, the compatibility of internal genes with porcine host cells and the synergistic interplay between internal and surface genes also influence the H9 subtype avian influenza virus’s ability to infect pigs. This insight offers a valuable framework for future comprehensive investigations.

## Supplementary Information

**Additional file 1. Sequence used in this study.**

**Additional file 2. Differences at the amino acid sequence level between H9 viruses capable and incapable of infecting pigs**. The amino acid sequences of H9 viruses were aligned, and the red box highlights differences between strains that are capable and incapable of infecting pigs. ST2030 and JX7554 were unable to infect pigs. NT449, NT10, SSP177W, and YU341 were infective to pigs. The numbering is in accordance with the H3 HA subtype. ST2030: A/Duck/Shantou/2030/2000, JX7554: A/Duck/Jiangxi/7554/2007, NT449: A/Chicken/Hong Kong/NT449/2007, NT10: A/Chicken/Hong Kong/NT10/2011, SSP177W: A/Chicken/Hong Kong/SSP177W/2009, Y341: A/Chicken/Hong Kong/YU341/2008.

**Additional file 3. Amino acid-differentiated sites on the HA monomer of H9 virus.** The positions with distinctive amino acids between viruses capable and incapable of infecting pigs are highlighted in yellow (in HA1), purple (in HA2) and red (amino acids with different properties). Fuchsia indicates the position of the connection peptide

## Data Availability

The genome sequences of A/Duck/Shantou/2030/2000, A/Duck/Jiangxi/7554/2007, and A/Chicken/Hong Kong/SSP177W/2009 are available in GenBank (accession numbers are listed in Additional file 1). The datasets generated and/or analyzed during the current study are included in this published article and its supplementary information files.
